# Analysis of plasma multiplex cytokines and increased level of IL-10 and IL-1Ra cytokines in febrile seizures

**DOI:** 10.1186/s12974-017-0974-7

**Published:** 2017-10-10

**Authors:** Kyungmin Kim, Byungok Kwak, Aram Kwon, Jongseok Ha, Soojin Kim, Sunwhan Bae, Jaesung Son, Soonyung Kim, Ran Lee

**Affiliations:** 1 Department of Pediatrics, Konkuk University Medical Center, Seoul, Korea; 2 Department of Pediatrics, Konkuk University Medical Center, Konkuk University School of Medicine, 120-1 Neungdong-ro (Hwayang-dong), Gwangjin-gu, Seoul, 05030 Korea; 3 Department of Obstetrics and Gynecology, Konkuk University Medical Center, Konkuk University School of Medicine, Seoul, Korea; 4 International Healthcare Research Institute, Konkuk University, Seoul, Korea

**Keywords:** Febrile seizures, Pro-inflammatory cytokines, Anti-inflammatory cytokines, IL-10, IL-1Ra

## Abstract

**Background:**

Febrile seizures are the most common form of childhood seizures. Fever generation involves many cytokines, including both pro- and anti-inflammatory cytokines. Some of these cytokines also induce febrile seizures. We compared cytokine production in children with a fever alone (healthy control group) and febrile seizure children group. Also, we evaluated the cytokine level of children with a fever alone and febrile seizure history.

**Methods:**

Fifty febrile seizure patients and 39 normal control patients who visited the emergency department of Konkuk University Hospital from December 2015 to December 2016 were included in this study. Blood was taken from the peripheral vessels of children in all groups within 1 h of the seizure, and serum was obtained immediately. Serum samples from patients with only a fever and a febrile seizure history (*N* = 13) and afebrile seizure controls (*N* = 12) were also analyzed.

**Results:**

The serum IL-10 and IL-1Ra levels were significantly higher in the febrile seizure patients than in the fever-only control, fever only with a febrile seizure history, and afebrile seizure groups (*p* < 0.05). The serum IFN-γ and IL-6 levels were significantly higher in the febrile seizure patients than in the afebrile seizure group (*p* < 0.05). The serum IL-8 levels were higher in the febrile seizure patients than in the fever only controls (*p* < 0.05).

**Conclusions:**

The serum levels of the IFN-γ, IL-6, and IL-8 pro-inflammatory cytokines and the serum levels of the IL-10 and IL-1Ra anti-inflammatory cytokines were significantly higher in the febrile seizure children. Furthermore, the serum level of IL-1Ra was more increased in the febrile seizure group than in the same patients with only a fever. Our data suggest that increased serum IL-10 and IL-1Ra may play potential roles as anti-inflammatory cytokines in a compensation mechanism that shortens the seizure duration or prevents a febrile seizure attack. Therefore, anti-inflammatory cytokines, including IL-10 and IL-1Ra, have potential as therapeutic targets for the prevention of seizures and nervous system development of children.

## Background

Febrile seizures (FSs) are the most common form of childhood seizures and occur in 2–5% of children before the age of 6 years [1]. FSs are seizures induced by fever (body temperature ≥ 38 °C). These patients do not have any definitive causative diseases, such as a central nervous system infection, electrolyte imbalance, metabolic disorders, and history of afebrile seizures [2]. Although several cohort studies have suggested that the prognosis of FSs is good, epilepsy is observed in 5.4% of these patients [3]. The exact pathogenesis of FSs is not well understood. However, predisposing factors, genetic susceptibility, infection or immune-mediated factors, and a cytokine storm have been proposed [4]. A positive family history of FSs is the most meaningful risk factor, with more affected relatives resulting in a greater risk [5]. Cytokines act as mediators of the host response to infections and induce fever, leukocytosis, and acute-phase protein synthesis [6]. Recently, abnormalities in cytokine and immune cell expression have been observed in patients with seizures and in animal models of seizures. Many studies have shown that the production and release of cytokines are regulated by the immune system and that cytokines can aggravate brain damage when acting as mediators of seizures [7]. Cytokines can exert both pro-inflammatory and anti-inflammatory effects [4]. Fever is induced by pro-inflammatory cytokines, such as interleukin (IL)-1β, IL-6, and tumor necrosis factor (TNF)-α, during infections. The fever threshold temperature for FSs varies among individuals as well as by age and maturation [8].

Gallentien WB et al. has measured multiple cytokines and compared the results between children with FSs and controls [9]. Due to recent advances in technology, a small amount of serum can be applied to multiplex flow cytometry to obtain multiple cytokine levels. In this study, we hypothesized that the cytokine profiles might differ between patients with FSs and control groups, such as individuals with only fever without a FS history, only fever with a FS history, and afebrile seizures. This result would suggest that pro-inflammatory and anti-inflammatory cytokines play roles in the pathogenesis of FSs. Therefore, we assessed the correlation between cytokine levels and FSs.

## Methods

### Patient information

Fifty febrile seizure patients and 39 normal control patients who visited the emergency department of Konkuk University Hospital, Seoul, Korea from December 2015 to December 2016 were included in this study. All parents were provided information regarding the research method. The children included in the study underwent blood tests after their parent agreed and signed the informed consent form.

The inclusion criteria for the febrile seizure groups were (1) age between 6 months and 6 years, (2) body temperature ≥ 38 °C, (3) C-reactive protein (CRP) < 3.0 mmol/dL, (4) presence of a simple febrile seizure (generalized seizure, usually tonic-clonic, lasting for < 15 min and not recurrent within a 24-h period), and (5) present no other identifiable cause of the seizure. Patients with a central nervous system infection, metabolic imbalance, and history of afebrile seizures were excluded. Laboratory findings, including complete blood counts (CBCs), blood chemistry, and CRP, were evaluated at the time of the seizure.

The control group included febrile children without seizures who visited our hospital for common febrile diseases, such as upper respiratory tract infections. Febrile diseases (viral or bacterial) were diagnosed according to the clinical manifestations. The control groups were matched for age and temperature criteria and had no convulsions during the febrile illness and no known history of previous febrile seizures.

Blood was taken from the peripheral vessels of children in all groups, and serum was obtained by centrifugation at 3500 rpm for 5 min at 4 °C. The serum was immediately separated and stored in a deep freezer at − 70 °C. In the febrile seizure groups, blood samples were collected within 1 h after the occurrence of a seizure episode.

Additionally, blood serum sample was collected and frozen from children with an afebrile seizure attack (*N* = 13) and a febrile illness with a febrile seizure history (*N* = 12) to subtract the effects of the fever from the cytokine levels.

The study was approved by the Institutional Review Board at the Konkuk University Medical Center (30 October 2015–30 October 2017).

### Cytokine measurement

The concentrations of pro-inflammatory cytokines (IFN-γ, IL-1β, IL-2, IL-6, and IL-8) and anti-inflammatory cytokines (IL-10 and IL-1Ra) were measured using commercially available enzyme-linked immunosorbent assay (ELISA) kits (MILLIPLEX MAP KIT, human cytokine/chemokine magnetic bead panel—immunology multiplex assay, Cat #HCYTOMAG-60 K, EMD Millipore Corp. Billerica, MA, USA). All samples were measured in duplicate to improve the accuracy.

### Statistical analysis

The data were summarized using the mean ± standard deviation (SD) for clinical characteristics, whereas the cytokine levels were expressed as the median and range. The chi-square test was used to compare the clinical findings, and the Kruskal–Wallis test or Mann–Whitney *U* test was performed to compare serum cytokine levels and laboratory findings among the FS, control, fever only with FS history, and afebrile seizure groups. Bonferroni correction was performed to account for multiple comparisons (corrected critical *p* value ≤ 0.05). Statistical calculations were performed using SPSS ver. 24. *p* < 0.05 was considered significant.

## Results

### Patient clinical profiles

Fifty patients with FSs and 39 healthy control children with febrile illness without seizures were included in this study. The demographic information and clinical characteristics of the children are summarized in Table 1. Although the mean age was younger in the FS group than in the control group (27.3 ± 13.2 vs. 36.3 ± 31.9 months), the difference was not significant (*p* = 0.327). The proportion of boys and girls (26/34 vs. 20/19) and the body temperature at admission were similar in the FS and control groups (38.9 ± 0.5 vs. 38.6 ± 0.6) (*p* < 0.05). The white blood cell (WBC) count was slightly increased in the control group compared to the FS group (10,861 ± 4296 vs. 12,758 ± 6023) (*p* = 0.143). The CRP was significantly higher in the control group than in the FS group (0.55 ± 0.7 vs. 3.6 ± 2.6) (*p* < 0.001). Furthermore, 13 children with fever only and with febrile seizure history and 12 children with afebrile seizures were included for comparison with the febrile seizure group. Boys were more prevalent than girls (8/4), and the CRP level was increased in the fever only with FS history group (4.8 ± 7.0). The mean age of the afebrile seizure patients was higher than the mean ages of the other groups (95.9 ± 60.8).

**Table 1:** Clinical findings of FS, control, fever only with FS history, and afebrile seizure groups

	Febrile seizure (*N* = 50)	Control (*N* = 39)	Fever only with FS history (*N* = 12)	Afebrile seizure (*N* = 13)	*p* value
Age (months)	27.3 ± 13.2	36.3 ± 31.9	40.5 ± 32.6	95.9 ± 60.8	0.327
Sex (male/female)	26/24	20/19	8/4	7/6	
BT (°C)	38.9 ± 0.5	38.6 ± 0.6	38.4 ± 0.5	36.8 ± 0.2	0.013
WBC (/μL)	10,861 ± 4,296	12,758 ± 6,023	10,642 ± 4,871	9750 ± 2,963	0.143
CRP (mg/dL)	0.55 ± 0.7	3.6 ± 2.6	4.8 ± 7.0	0.13 ± 0.1	< 0.001

Data are presented as mean ± SD (standard deviation). The *p* value is about FS and control groups. A *p* value < 0.05 indicates a significant difference. Mann–Whitney *U* test

*FS* febrile seizure, *BT* body temperature, *WBC* whole blood cell, *CRP* C-reactive protein

### Comparison of cytokines

A comparison of the plasma cytokines in the four groups (FS, febrile control, fever only with FS history and afebrile seizure) is shown in Table 2. IFN-γ, IL-10, IL-1Ra, IL-2, IL-6, and IL-8 were increased in the FS group (*p* = 0.047, <0.001, <0.001, 0.007, 0.001, and 0.030, respectively; Table 2). The remaining cytokines except IL-1β and IL-2 were significantly increased in the FS group when subjected to the Bonferroni multiple comparison correction test. The mean IFN-γ level was 48.3 pg/mL in the FS group and 13.7 pg/mL in the afebrile seizure group. Comparisons of the IFN-γ levels showed significantly higher levels in the FS group than in the only afebrile seizure group (corrected *p* = 0.029). The mean IL-10 level was 156.9 pg/mL in the FS group and was significantly increased compared to the other three groups (febrile control, fever only with FS history and afebrile seizure) and had a significant corrected *p* value within each group comparison. The IL-1Ra level was also higher in the FS group compared to the other three groups, with corrected *p* values for each group comparison below 0.05. The mean IL-1β level was 3.8 pg/mL in the FS group and 4.5 pg/mL in the febrile control group. Comparisons of the IL-1β levels among the four groups showed no significant differences (*p* = 0.300, Table 3). The mean IL-2 level was 3.0 pg/mL in the FS group and 3.3 pg/mL in the febrile control group. The *p* value was significantly lower when the IL-2 levels were compared among the four groups; however, this result was due to a difference between the afebrile seizure and febrile control group and did not affect the FS group. The mean IL-8 concentration was 109.1 pg/mL in the FS group and 33.8 pg/mL in the febrile control group. Comparisons of the IL-8 levels showed an increase in the FS group compared with the febrile control group (corrected *p* = 0.042). Interestingly, we also compared the serum cytokine levels between the same patients after a febrile seizure and when they had only a fever without a seizure (Table 3). The median IL-1Ra level was 638.9 pg/mL during febrile seizes and 177.6 pg/mL when the same patients only had a fever. The *P* value was less than 0.05 and was significantly increased in the FS group. The median IL-2 level was 2.86 pg/mL in the febrile seizure group and 1.83 pg/mL in the same patients with only a fever. Comparison of the IL-2 levels between the FS and fever-only conditions showed a significant increase in the FS condition (*p* = 0.029, Table 3). The remaining cytokines excluding IL-1Ra and IL-2 showed no differences in their levels in the FS condition.

**Table 2:** Comparisons of cytokine levels in four groups including FS, control, fever only with FS history, and afebrile seizure children

Cytokines	Febrile seizure (*N* = 50)	Control (*N* = 39)	Fever only with FS history (*N* = 12)	Afebrile seizure (*N* = 13)	*p* value
IFN-γ	29.8(4–220)	21.6(2.7–703)	21(5.0–266)	13.9(0.3–25.8)	0.047
IL-10	103.7(7.1–728)	34.0(1.4–832.4)	22.0(0.5–181)	5.0(0.3–65.3)	< 0.001
IL-1Ra	652(144–5667)	231(1.6–1461)	202(34–422)	200(0.3–3440)	< 0.001
IL-1β	2.8(2.8–16.2)	2.9(0.4–47.4)	2.2(0.4–25.4)	1.1(0.2–61.6)	0.300
IL-2	2.8(2.8–5.3)	2.8(0.3–17.1)	1.7(0.2–27.2)	0.9(0.1–42.2)	0.007
IL-6	16.9(1.3–798)	7.9(0.2–104.2)	6.5(0.2–71.4)	0.8(0.2–31.5)	0.001
IL-8	43.3(2.3–1711)	20.9(1.3–109.1)	35.5(2.6–589)	54.7(4.5–345.6)	0.030

Data are presented as median (range), and the unit is pg/mL. Multiple comparison test (Bonferroni correction). A *p* value < 0.05 indicates a significant difference. Kruskal–Wallis test

**Table 3:** Comparison of cytokine levels between febrile seizures and fever only in four patients

	Febrile seizure (*N* = 4)	Fever only with FSs history (*N* = 4)	*p* value
IFN-γ	68.4(28.7–121.7)	21.5(8.39–161.49)	0.486
IL-10	50.8(40.1–84.5)	17.7(4.3–181.5)	0.343
IL-1Ra	638.9(478–1244)	177.6(40.9–281.7)	0.029
IL-1β	3.4(2.8–4.7)	1.88(1.2–3.9)	0.114
IL-2	2.86(2.8–3.6)	1.83(0.4–2.2)	0.029
IL-6	17.27(6.4–96.5)	4.43(0.4–7.6)	0.057
IL-8	27.2(11.4–59.4)	39.6(14.9–144.1)	0.486

Four patients of both groups are same children. Data are presented as median (range) and unit is pg/mL. A *p* value < 0.05 indicates a significant difference. Mann–Whitney *U* test

## Discussion

Although many studies have investigated the cause of febrile seizures, the actual cause of the disease is mostly unknown. Few studies have suggested the role of cytokines in febrile seizures [10]. Since FSs occur during the course of a high body temperature or a rapidly rising fever in susceptible individuals, various factors associated with fever generation could be involved in the possible causative mechanism of FSs. Fever generation involves many cytokines and endogenous mediators, including IL-1, IL-6, TNF-α, IL-1Ra, and IL-10, in response to exogenous pyrogens. Peripheral cytokines can impact brain functions as evidenced by CNS symptoms, such as fever, anorexia, lethargy, and a lower seizure threshold, during systemic inflammation, febrile illness, or sepsis. Although peripheral cytokine levels may not be an accurate reflection of CNS cytokine activity, there are mechanisms by which their presence may induce a reciprocal inflammatory response within the brain [11]. Several studies showed blood–brain barrier (BBB) failure after administration of IL-1, IL-6, TNF-α, and IFN-γ. This inflammation induces changes in the brain parenchyma, such as leakage of the BBB, which changes the functional properties of the BBB [12]. These changes in BBB permeability may favor the entry of peripheral cytokines as well as cells of the innate and adaptive immune response into the CNS, resulting in further activation of the inflammatory cascade within the brain [11]. The changes cause cell damage that contributes to neuronal hyperexcitability, which lowers the threshold for seizure induction and triggers epileptogenesis [12].

Another study reported that the plasma IL-1β and cerebrospinal fluid TNF-α levels were significantly higher in FS patients during the acute phase of the disease than in the controls [8, 13]. The chronic IL-1β expression during epileptogenesis highlights the possibility that this cytokine may be involved in the mechanisms underlying the onset of spontaneous seizures [14]. Gallentine WB et al. compared the samples of children with febrile status epilepticus (FES) to children with fever. They found that as the balance shifts toward higher IL-1β and lower IL-1Ra, seizure threshold goes down and the likelihood of FSE increases. On the contrary, as the balance shifts toward lower IL-1β and higher IL-1Ra, seizure threshold is higher and the likelihood of FSE decreases [9].

In the present study, we analyzed a total of seven cytokines, including pro-inflammatory and anti-inflammatory cytokines, as described above. The multiplex cytokine analysis revealed no increase in serum IL-1β in our children with FSs compared to the controls. Therefore, in contrast to previous studies, these results conflict with the important role played by IL-1β in the mechanism of febrile seizures. This lack of an increase may be the result of excluding patients with presumptive bacterial infections, such as a low CRP level, because LPS is the main inducer of IL-1β synthesis [15]. Moreover, IL-1β is usually difficult to detect due to its binding to large proteins, such as α-2 microglobulin and complement. Furthermore, fever can occur independently of IL-1β activity during infections, possibly through the cytokine-like property of Toll-like receptor signal transduction [16]. The contradictory results reported by various studies may be due to the interference of confounding variables, such as time of sampling, severity of temperature, duration of fever, difficulty in measuring cytokines, type of infection, and sample size. Time of sampling is very important for measurement of the IL-1β levels. IL-1β increases within 1 h after a seizure, reaches its maximum level within 4–12 h after a seizure, and returns to its normal level after 24 h [17]. Thus, IL-1β is best measured within 12 h after the incidence of seizure. In the present study, samples were prepared within 1 h after the incidence of seizure. Therefore, in this study, low levels of IL-1β in patients with febrile seizures did not correlate with the time of sampling.

During the response to seizures, the IL-1β system induces IL-1Ra, which acts by limiting IL-1β-mediated pro-inflammatory actions. IL-1Ra has been shown to be a powerful anticonvulsant in various seizure models. IL-1Ra is induced by seizures several hours after IL-1β to rapidly terminate the effects of IL-1β. In another study, peak pro-inflammatory cytokine effects occurred 6 h after status epilepticus, whereas the peak effect of the anti-inflammatory cytokine IL-1Ra was delayed and occurred 24 h after IL-1β [18]. Therefore, IL-1Ra is induced by seizures several hours after IL-1β [19]. Changes in the IL-1Ra to IL-1 ratio act as a mechanism to control seizures after onset. During peripheral inflammatory reactions, IL-1Ra is generally produced at a 100-fold molar excess compared to IL-1 [14]. This change may be an effective pathophysiological mechanism to control seizures. In our study, IL-1Ra was significantly higher in the FS group than in the control, fever only with FS history, and afebrile seizure groups. This is contrary to Gallentine WB et al. that the IL-1Ra was not increased in FSE when comparing FSE to control patients. Because IL-1Ra has neuroprotective and anticonvulsant effects [20] and is closely correlated with the pro-convulsive cytokine IL-1β, we suggest that elevated IL-1Ra may be hyperreactive to IL-1β in response to the epileptogenic environment even though the IL-1β level does not change significantly. The IL-1β level is expected to react faster than the time presented in previous studies and will decrease in the usual range. Interestingly, the serum IL-1Ra level was also increased when we compared the cytokines between the same four patients in the presence of a febrile seizure and with a fever alone without a febrile seizure. Therefore, IL-1Ra is expected to increase the anti-inflammatory reaction and reduce the febrile convulsion duration and thus seems to be a more accurate indicator than IL-1β.

IL-6 is a cell signaling molecule that has been associated with many diseases, including inflammatory, neurological, vascular, and malignant processes [21]. The systemic concentration of IL-6 is mainly regulated at the level of expression, because IL-6 is rapidly cleared from the plasma and has a short plasma half-life of 20–60 min [22]. Many other studies have supported the potential immune modulatory effects of IL-6 and its importance as a major pro-inflammatory cytokine. The plasma IL-6 level was significantly higher in patients with FSs compared with the febrile control subjects, and the CSF IL-6 levels were detectable in all studied patients with FSs [23]. As expected, the current study showed that the serum IL-6 levels were markedly elevated in the patients with FSs compared to the afebrile seizure group. Our results together with other findings may support the proconvulsant action of IL-6 in FSs.

IL-10 is a multifunctional anti-inflammatory cytokine that is produced by monocytes, macrophages, lymphocytes, and microglia and inhibits the production of pro-inflammatory cytokines, including IL-1, IL-6, IL-8, and TNF-α [24]. Previous studies have shown lipopolysaccharide-induced increases in IL-10 production by peripheral blood mononuclear cells isolated from children with a history of FSs. Another group reported that the plasma IL-10 level did not differ between children with and without FSs. Therefore, little is known about the involvement of IL-10 in the pathogenesis of FSs [23]. However, the febrile seizure threshold temperatures were significantly higher in mice injected with recombinant human IL-10 to establish a hyperthermia-induced seizure model than in the control group [25]. As expected, in the present study, the IL-10 levels were higher in the FS group than in control, fever only with FS history, and afebrile seizure groups. These findings may reflect compensatory activation of the anti-inflammatory role or an anti-convulsive mechanistic effect of IL-10 in FSs.

Interestingly, IL-8 was also elevated in our study. IL-8 exhibits trophic-like activity and is implicated in the maintenance of normal neuronal populations as well as the promotion of neuronal survival and regeneration. IL-8 is also a known neutrophil activation peptide and is produced by monocyte-derived macrophages, microglia, and astrocytes [26]. IL-8 strongly promotes leukocyte migration to inflammatory foci in the CNS by inducing neutrophil–endothelial adhesions and contributes to BBB breakdown during acute brain injury. Therefore, the present study suggests that IL-8 may contribute to the proconvulsant environment and possibly promote epileptogenesis.

IL-2 plays an important role in regulating the immune response by driving T cell growth, augmenting NK cytolytic activity, inducing the differentiation of regulatory T cells, and mediating activation-induced cell death [27]. Intraventricular administration of IL-2 in DBA/2 mice promotes seizure generation in various models of experimental epilepsy [28]. However, the IL-2 levels in the plasma and CSF remained unchanged in patients with prolonged FSs with or without encephalopathy during the acute stage [29]. As shown in our results, the IL-2 dose level did not significantly differ between the children with FSs and the control group. However, the serum IL-2 level was increased when we compared serum cytokines between the same four patients after a febrile seizure and when there was fever only without a febrile seizure. Although the number of patients in the case is small, we suggest that IL-2 is also correlated with febrile seizures as a pro-inflammatory cytokine.

## Conclusions

In conclusion, pro-inflammatory cytokines, including IFN-γ, IL-6, and IL-8 and anti-inflammatory cytokines, including IL-1Ra and IL-10, were increased in children with FSs. Furthermore, the serum level of IL-1Ra was more increased in the febrile seizure group than in the same patients with only a fever. If pro-inflammatory cytokines are produced by the IL-1 systemic cascade in the febrile condition, peripheral cytokines enter the CNS and lower the seizure threshold. Pro-inflammatory cytokine production may promote seizures, further exacerbate epilepsy, and cause subsequent intractable epilepsy. Anti-inflammatory cytokines were also elevated in children with FSs as a compensatory mechanism to prevent FS attacks. The symptom of seizures improved within 5 min in almost all of the children with FSs due to the effects of anti-inflammatory cytokines. Therefore, anti-inflammatory cytokines, including IL-10 and IL-1Ra, may play critical roles and represent therapeutic targets for the prevention of seizures and for nervous system development in children.

There are two reasons why our research results, which are different from other studies, are more meaningful. First, we selected both a group of healthy children accompanied only by fever and a group of children with a fever only and a FS history to compare the cytokine levels. Second, by comparing the levels of cytokines during an FS episode and during fever alone in the same patient, various factors that occurred when comparing different patients could be eliminated. Although the number of samples compared in the same patient was small, the results supported the results compared with the fever-only healthy children control group. Subsequently, by further increasing the number of samples, other cytokines related to FSs could be clearly identified, especially IL-10 and IL-1Ra.

## Abbreviations

BBB: Blood–brain barrier
CBC: Complete blood count
COX: Cyclooxygenase
CRP: C-reactive protein
FS: Febrile seizure
IL: Interleukin
LPS: Lipopolysaccharide
PGE: Prostaglandin E
TNF: Tumor necrosis factor
WBC: Whole blood cell
