# Correction to: Analysis of plasma multiplex cytokines and increased level of IL-10 and IL-1Ra cytokines in febrile seizures

**DOI:** 10.1186/s12974-017-0986-3

**Published:** 2017-11-17

**Authors:** Kyungmin Kim, Byung Ok Kwak, Aram Kwon, Jongseok Ha, Soo-Jin Kim, Sun Whan Bae, Jae Sung Son, Soo-Nyung Kim, Ran Lee

**Affiliations:** 1 Department of Pediatrics, Konkuk University Medical Center, Seoul, South Korea; 2 Department of Pediatrics, Konkuk University Medical Center, Konkuk University School of Medicine, 120-1 Neungdong-ro (Hwayang-dong), Gwangjin-gu, Seoul, 05030 South Korea; 3 Department of Obstetrics and Gynecology, Konkuk University Medical Center, Konkuk University School of Medicine, Seoul, South Korea; 4 International Healthcare Research Institute, Konkuk University, Seoul, South Korea

## Correction

After publication of the article [1], it has been brought to our attention that several of the authors’ names were formatted incorrectly in the original version of the article. The corrections are listed below –

“Byungok Kwak” should be “Byung Ok Kwak”“Soojin Kim” should be “Soo-Jin Kim”“Sunwhan Bae” should be “Sun Whan Bae”“Jaesung Son” should be “Jae Sung Son”“Soonyung Kim” should be “Soo-Nyung Kim”

The original version of the article has now been revised.
